# Patient-reported disease burden and health care utilization of HAE-nl-C1INH: insights from a real-world survey

**DOI:** 10.1007/s10238-025-02010-5

**Published:** 2026-01-14

**Authors:** Douglas Jones, Nihal Narsipur, Sally W. Wade, Joseph R. Harper, Nami Park, Philippe Adams, Anurag Relan, Amanda Harrington, John Anderson

**Affiliations:** 1 Rocky Mountain Allergy, Asthma, and Immunology, Salt Lake City, UT USA; 2 Pharming Healthcare, Inc., Warren, NJ USA; 3 Wade Outcomes Research and Consulting, Salt Lake City, UT USA; 4 AllerVie Health, Birmingham, AL USA; 5 University of Alabama at Birmingham, Birmingham, AL USA

**Keywords:** Hereditary angioedema, HAE-nl-C1INH, Disease burden, Health care resource utilization, Health-related quality of life, HAE disease characteristics

## Abstract

Hereditary angioedema (HAE), a rare genetic disorder, is classified into 3 types: Type 1 (low C1 esterase inhibitor [C1-INH]), Type 2 (dysfunctional C1-INH), and HAE-nl-C1INH (normal C1-INH levels). This study aimed to compare characteristics among individuals with HAE Type 1/2 and HAE-nl-C1INH. A cross-sectional online survey was conducted (June 2020–September 2021) among adults with HAE to capture various patient-specific data and health care utilization. Statistical analyses included Fisher exact test, *t* test, and odds ratios (OR) with 95% confidence intervals (CI). Eighty-nine participants were included (HAE Type 1/2, *n* = 44; HAE-nl-C1INH, *n* = 45). No significant differences in demographics, treatment characteristics, and HAE triggers were observed between groups. Participants with HAE-nl-C1INH were less likely to be diagnosed before 18 years of age (4% vs. 32%; OR, 0.10; 95% CI, 0.02–0.50) and had higher odds of experiencing frequent HAE attacks (47% vs. 14%; OR, 5.5; 95% CI, 2.0–15.7) than those with HAE Type 1/2. Participants with HAE-nl-C1INH also had increased odds of orofacial-laryngeal swelling (31% vs. 16%; OR, 2.3; 95% CI, 1.1–4.7), more frequent doctor visits (> 1 visit/month: 31% vs. 11%; OR, 3.5; 95% CI, 1.1–10.8), and more concomitant conditions (93% vs. 77%; OR, 3.8; 95% CI, 1.6–9.2). The total health-related quality of life score was significantly worse in participants with HAE-nl-C1INH (53.6 vs. 38.2; *p* = 0.0001). Participants with HAE-nl-C1INH experience a significantly greater disease burden than those with HAE Type 1/2, emphasizing the need for improved diagnosis, targeted treatment strategies, and a deeper understanding of the prevalence and pathophysiology of HAE-nl-C1INH.

## Introduction

Hereditary angioedema (HAE) is a rare genetic disorder affecting an estimated 1 in 50,000 people globally [1]. Unpredictable episodes of swelling at various anatomical locations, including the extremities, abdomen, genitourinary tract, and orofacial-laryngeal areas, are associated with HAE [1, 2]. HAE attacks that target the upper airways can be potentially life-threatening. Swelling in the hands and feet can impair function, and abdominal attacks can be associated with significant pain [1, 3]. The debilitating and unpredictable nature of this disease can decrease participation in daily activities, increase health care resource use, and negatively impact the health-related quality of life (HRQoL) of patients [4, 5].

Unlike allergic angioedema, HAE is not mediated by histamine but by bradykinin, a potent vasodilator [6, 7]. HAE is usually caused by genetic variants affecting C1 esterase inhibitor (C1-INH); deficient or dysfunctional C1-INH leads to unrestrained activation of the kallikrein-kinin system, resulting in augmented bradykinin levels, vascular permeability, and localized swelling [6, 7]. HAE is generally classified into 3 types: Type 1, characterized by a low level of C1-INH; Type 2, characterized by present but dysfunctional C1-INH; and HAE-nl-C1INH, characterized by normal levels of C1-INH and clinical features that are similar to HAE Types 1 and 2 [1, 2]. HAE Types 1 and 2 are typically grouped under the term HAE-C1-INH, distinguishing a lower level and/or functionality of the C1-INH protein compared with HAE-nl-C1INH [8]. Although HAE-nl-C1INH was described in 2000, its prevalence and pathophysiology are not clearly defined [1, 2]. In clinician-reported survey data, approximately 77% to 81% and 19% to 23% of patients with HAE were estimated to have HAE Type 1/2 and HAE-nl-C1INH, respectively, with an estimated prevalence of 0.37 per 100,000 people in the US [9].

Although these 3 distinct HAE types reflect the nature of C1INH levels and/or function, little has been reported about the specific characteristics and experiences of HAE-nl-C1INH compared with the more prevalent HAE Types 1 and 2 [1, 8, 10]. Consequently, HAE guidelines provide limited information on the characteristics and experiences associated with specific HAE types [1, 10, 11]. Therefore, the objective of this cross-sectional online survey was to evaluate and compare the characteristics of patients with HAE Types 1 and 2 (HAE Type 1/2) with those of patients with HAE-nl-C1INH.

## Methods

A voluntary, cross-sectional online survey was conducted between June 16, 2020, and September 3, 2021. Adult patients with HAE who were referred to a patient support program associated with their prescription HAE treatment were invited to participate in the study. Participants were excluded from the survey if they were younger than 18 years, had limited English reading or comprehension skills, or did not report their HAE category (i.e., Type 1, Type 2, HAE-nl-C1INH). Prior to survey initiation, the protocol and survey were reviewed by Advarra Institutional Review Board (Columbia, MD), and an exemption from institutional review board oversight was granted. All participants provided informed consent upon entry to the online survey.

Participants had 2 weeks to complete the survey, which was immediately accessible to participants after completing the agreement to participate. The survey consisted of multiple-choice questions, free-response questions, and rank-order questions that used a symmetrical 5- to 7-point Likert scale of agreement for scale-based responses.

Collected data included participant characteristics and demographics (e.g., general medical and HAE history), symptoms (e.g., frequency and nature of HAE attacks), participant-reported outcomes (e.g., HRQoL, depression, and anxiety), and HAE-related health care utilization. The US HAE Association Quality of Life Questionnaire, a 27-item instrument specific to patients with HAE, was used to assess HRQoL [12].

Categorial variables were described using n (%) and compared using the Fisher exact test. Continuous variables were described using mean (standard deviation [SD]) and compared using the *t* test. To estimate the magnitude of association, odds ratios (OR) with 95% confidence intervals (CI) were reported for response categories with a statistically significant difference between HAE-nl-C1INH and HAE Type 1/2. Cohen *d*, also known as the standardized mean difference, was used to classify the effect size between groups; 0.2, 0.5, and 0.8 indicated small, moderate, and substantial effect sizes, respectively.

The statistical software used was Python, version 3.12.4. All figures were created in PowerPoint.

## Results

### Baseline characteristics

**Fig. 1:**
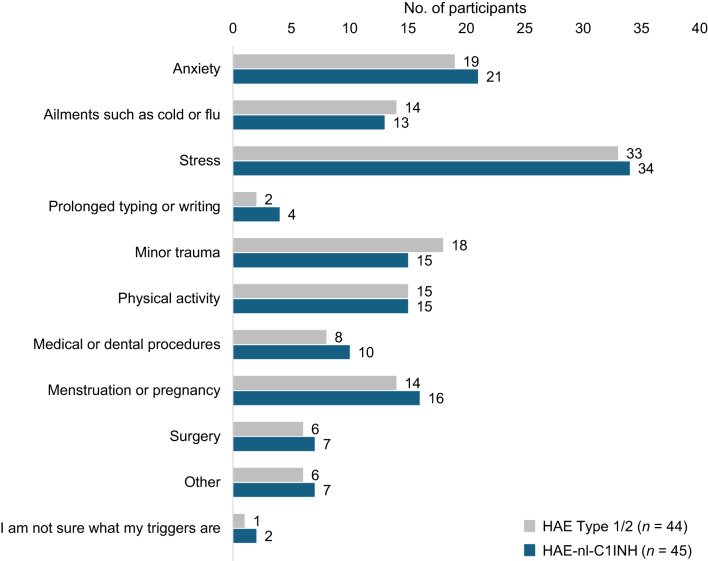
Most common HAE triggers. HAE, hereditary angioedema; HAE-nl-C1INH, hereditary angioedema with normal levels of C1 esterase inhibitor

Among the 89 eligible participants who completed the survey, most were women (90%; 80 of 89), White or Caucasian (78%; 69 of 89), and between 31 and 50 years of age (53%; 47 of 89). There were no significant differences in demographic characteristics (Table 1), treatment characteristics (Table 2), or HAE triggers (Fig. 1) between participants with HAE-nl-C1INH and HAE Type 1/2 (all *p* > 0.05). Across groups, stress was the most common trigger, reported in 75% (67 of 89) of participants.

**Table 1:** Respondent demographics

Category	HAE Type 1/2 (*n* = 44)	HAE-nl-C1INH (*n* = 45)	Overall population (*n* = 89)
**Patient gender, No. (%)**			
Female	37 (84)	43 (96)	80 (90)
Male	7 (16)	2 (4)	9 (10)
**Patient race or ethnicity**,** No. (%)**			
White or Caucasian	35 (80)	34 (76)	69 (78)
Black or African American	3 (7)	5 (11)	8 (9)
Hispanic or Latino origin	3 (7)	4 (9)	7 (8)
Asian or Pacific Islander	3 (7)	0	3 (3)
Mixed race/ethnicity	0	2 (4)	2 (2)
Native American or Alaskan	0	0	0
**Patient age**,** No. (%)**			
18–30 y	7 (16)	10 (22)	17 (19)
31–40 y	12 (27)	15 (33)	27 (30)
41–50 y	13 (30)	7 (16)	20 (22)
51–60 y	8 (18)	6 (13)	14 (16)
61–70 y	4 (9)	4 (9)	8 (9)
71–80 y	0	3 (7)	3 (3)
> 80 y	0	0	0

HAE, hereditary angioedema; HAE-nl-C1INH, hereditary angioedema with normal levels of C1 esterase inhibitor

**Table 2:** Treatment characteristics^a, b^

Category	HAE Type 1/2 (*n* = 55 responses)	HAE-nl-C1INH (*n* = 56 responses)
Medication used to treat active HAE swell, No. (%)		
Icatibant injection [FIRAZYR^®^]	24 (44)	16 (29)
C1 esterase inhibitor (human) [CINRYZE^®^]	3 (5)	2 (4)
C1 esterase inhibitor, human [BERINERT^®^]	5 (9)	3 (5)
Ecallantide [KALBITOR^®^]	1 (2)	3 (5)
C1 esterase inhibitor (recombinant) [RUCONEST^®^]	10 (18)	17 (30)
Newly diagnosed/have no medication	12 (22)	15 (27)

Category	HAE Type 1/2 (***n*** = 46 responses)	HAE-nl-C1INH (***n*** = 47 responses)
Medication currently used to prevent HAE swells, No. (%)		
I use on-demand treatment only	9 (20)	12 (26)
Lanadelumab-flyo [TAKHZYRO^®^] injection	8 (17)	7 (15)
C1 esterase inhibitor subcutaneous (human) [HAEGARDA^®^]	5 (11)	4 (9)
C1 esterase inhibitor subcutaneous (human) [CINRYZE^®^]	1 (2)	2 (4)
Newly diagnosed/have no medication	23 (50)	22 (47)

^a^Sum of the number of participant responses may not equal the number of participants as some participants may not have answered the question, and multiple responses were permitted for some questions. The sum of the percentages may not be equal to 100% due to rounding

^b^Participants responded to questions at the time of the survey. There are no participant data related to initiation or duration of treatment

HAE, hereditary angioedema; HAE-nl-C1INH, hereditary angioedema with normal levels of C1 esterase inhibitor

### Age at diagnosis of HAE

**Fig. 2:**
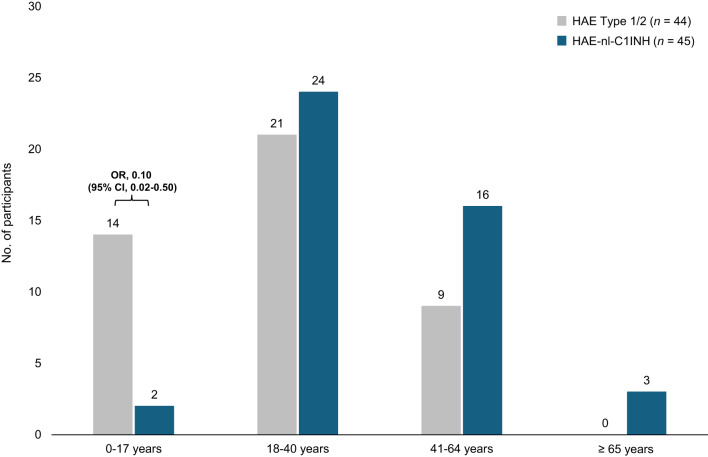
Age at diagnosis of HAE. CI, confidence interval; HAE, hereditary angioedema; HAE-nl-C1INH, hereditary angioedema with normal levels of C1 esterase inhibitor; OR, odds ratio

Participants with HAE Type 1/2 were more likely to have been diagnosed at a younger age compared with those with HAE-nl-C1INH (Fig. 2). 32% (14 of 44) of participants with HAE Type 1/2 were diagnosed before 18 years of age, compared with 4% (2 of 45) of those with HAE-nl-C1INH (OR, 0.10; 95% CI, 0.02–0.50).

### Concomitant conditions in participants with HAE

**Fig. 3:**
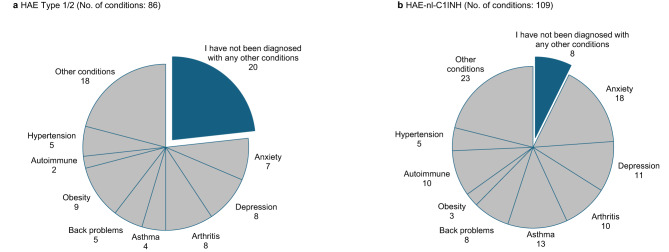
Concomitant conditions in participants with (**a**) HAE Type 1/2 HAE or (**b**) HAE-nl-C1INH. Other conditions reported by participants with HAE Type 1/2 or HAE-nl-C1INH included cancer (*n* = 3 and *n* = 2, respectively), cardiovascular disease (*n* = 0 and *n* = 2, respectively), chronic obstructive pulmonary disease (*n* = 0 and *n* = 1, respectively), diabetes (*n* = 2 and *n* = 2, respectively), lupus (*n* = 2 and *n* = 2, respectively), mental health conditions (*n* = 3 and *n* = 4, respectively), and conditions not otherwise specified (*n* = 8 and *n* = 10, respectively). HAE, hereditary angioedema; HAE-nl-C1INH, hereditary angioedema with normal levels of C1 esterase inhibitor

Eighty-six concomitant conditions were reported by participants with HAE Type 1/2, compared with 109 concomitant conditions reported in participants with HAE-nl-C1INH (Fig. 3). Participants with HAE-nl-C1INH were more likely to report concomitant conditions (OR, 3.8; 95% CI, 1.6–9.2) than those with HAE Type 1/2 (93% vs. 77%, respectively; *p* = 0.002). Commonly reported concomitant conditions across groups were anxiety, depression, arthritis, asthma, back problems, obesity, autoimmunity, and hypertension.

### Swelling location in the past 3 months

**Fig. 4:**
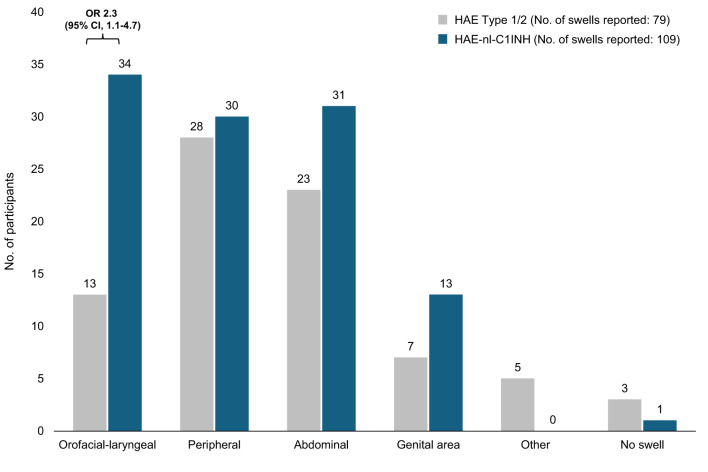
Swell location in the past 3 months. CI, confidence interval; HAE, hereditary angioedema; HAE-nl-C1INH, hereditary angioedema with normal levels of C1 esterase inhibitor; OR, odds ratio

In the 3 months before the start of the study, more swells were reported by participants with HAE-nl-C1INH than by participants with HAE Type 1/2 (109 vs. 79, respectively) (Fig. 4). Participants with HAE-nl-C1INH reported numerically more peripheral, abdominal, and genital area swells than those with HAE Type 1/2 and were more likely to report orofacial-laryngeal (i.e., face, tongue, and throat) swells (OR, 2.3; 95% CI, 1.1–4.7) compared with participants with HAE Type 1/2 (31% vs. 16%, respectively) despite similar participant counts.

### Frequency of HAE attacks in past 3 months

**Fig. 5:**
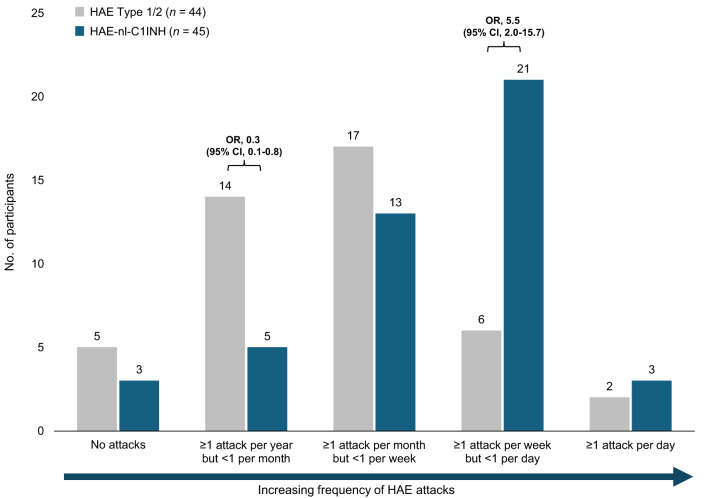
Frequency of HAE attacks in the past 3 months. CI, confidence interval; HAE, hereditary angioedema; HAE-nl-C1INH, hereditary angioedema with normal levels of C1 esterase inhibitor; OR, odds ratio

Participants with HAE-nl-C1INH reported more frequent HAE attacks in the 3 months prior to the study compared with those with HAE Type 1/2 (Fig. 5). Participants with HAE-nl-C1INH were more likely to report experiencing an HAE attack several times per week but less than once per day (OR, 5.5; 95% CI, 2.0–15.7) than those with HAE Type 1/2 (47% vs. 14%, respectively). Conversely, the odds of reporting an HAE attack more than once per year but less than once per month were 70% lower (OR, 0.3; 95% CI, 0.1–0.8) among participants with HAE-nl-C1INH than those with HAE Type 1/2 (11% vs. 32%, respectively).

### Frequency of doctor visits for HAE

**Fig. 6:**
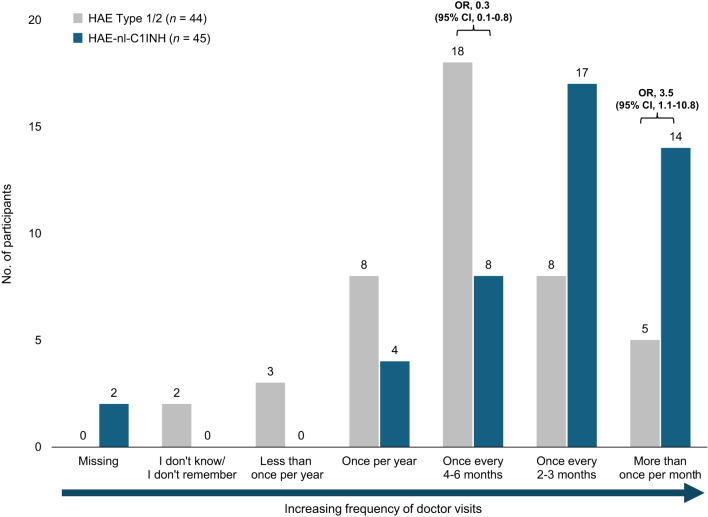
Frequency of doctor visits for HAE. CI, confidence interval; HAE, hereditary angioedema; HAE-nl-C1INH, hereditary angioedema with normal levels of C1 esterase inhibitor; OR, odds ratio

Participants with HAE-nl-C1INH reported more frequent doctor visits than participants with HAE Type 1/2 (Fig. 6). Participants with HAE-nl-C1INH were more likely to report visiting the doctor more than once a month (OR, 3.5; 95% CI, 1.1–10.8) than those with HAE Type 1/2 (31% vs. 11%, respectively) (Fig. 6). Conversely, a 70% odds reduction (OR, 0.3; 95% CI, 0.1–0.8) was observed in participants with HAE-nl-C1INH who reported less frequent doctor visits (once every 4–6 months) than participants with HAE Type 1/2 (18% vs. 41%, respectively).

**Fig. 7:**
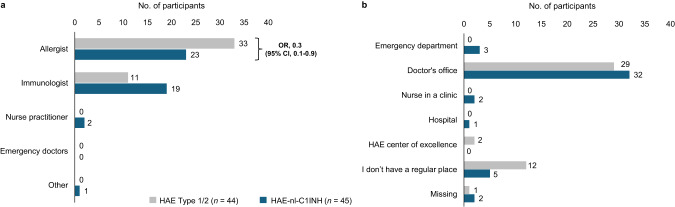
(**a**) Doctor specialty and (**b**) site of care for participants with HAE. CI, confidence interval; HAE, hereditary angioedema; HAE-nl-C1INH, hereditary angioedema with normal levels of C1 esterase inhibitor; OR, odds ratio

The odds of requiring a visit or seeking care from an allergist were 70% lower among participants with HAE-nl-C1INH (OR, 0.3; 95% CI, 0.1–0.9) than those with HAE Type 1/2 (51% vs. 75%, respectively) (Fig. 7a). Additionally, the percentage of participants with HAE-nl-C1INH who sought care from an immunologist was numerically higher than that of participants with HAE Type 1/2 (42% vs. 25%, respectively). A numerically higher proportion of participants with HAE Type 1/2 did not have a regular site of care compared with participants with HAE-nl-C1INH (27% vs. 11%, respectively) (Fig. 7b).

### Quality of life measures

**Fig. 8:**
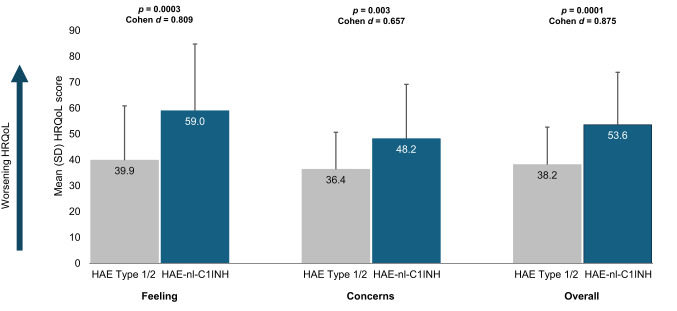
US HAE Association Quality of Life Questionnaire [12] scores. HAE Type 1/2 (*n* = 44) and HAE-nl-C1INH (*n* = 45). Cohen *d* interpretation: 0.2, small effect size; 0.5, moderate effect size; 0.8, substantial effect size. HAE, hereditary angioedema; HAE-nl-C1INH, hereditary angioedema with normal levels of C1 esterase inhibitor; HRQoL, health-related quality of life; SD, standard deviation

Participants with HAE-nl-C1INH reported significantly worse HRQoL than those with HAE Type 1/2 (Fig. 8). Participants with HAE-nl-C1INH had substantially higher mean HAE HRQoL scores than HAE Type 1/2 participants for feeling domains (59.0 vs. 39.9; *p* = 0.0003; Cohen *d* = 0.809) and moderately higher scores for concerns domains (48.2 vs. 36.4; *p* = 0.003; Cohen *d* = 0.657). Participants with HAE-nl-C1INH had substantially higher overall mean HAE HRQoL score (53.6 vs. 38.2; *p* = 0.0001; Cohen *d* = 0.875).

**Fig. 9:**
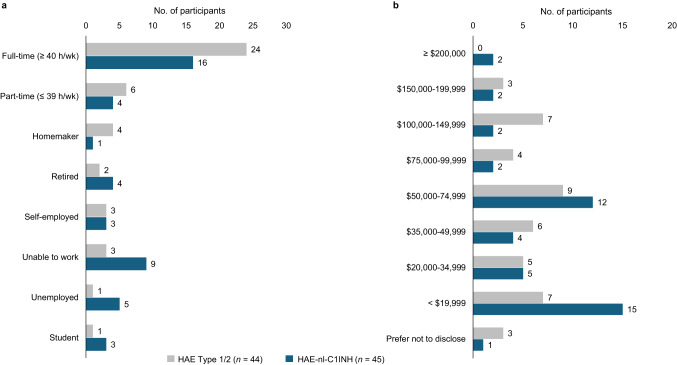
Employment status (**a**) and income (**b**) in participants with HAE. HAE, hereditary angioedema; HAE-nl-C1INH, hereditary angioedema with normal levels of C1 esterase inhibitor

The percentage of participants who reported being unable to work full time was numerically lower in those with HAE-nl-C1INH than in those with HAE Type 1/2 (36% vs. 55%, respectively), and the percentage who reported being unable to work was numerically higher among those with HAE-nl-C1INH than those with HAE Type 1/2 (20% vs. 7%, respectively) (Fig. 9a). In addition, numerically more participants with HAE-nl-C1INH reported earning less than $19,999 per year than those with HAE Type 1/2 (33% vs. 16%, respectively) (Fig. 9b).

## Discussion

The findings from this cross-sectional survey underscore key differences in disease burden and patient experiences between individuals with HAE Type 1/2 and those with HAE-nl-C1INH. While demographic characteristics were similar across groups, participants with HAE-nl-C1INH exhibited significantly greater disease burden in terms of attack frequency, anatomical distribution of swelling, health care utilization, and overall HRQoL.

One of the most striking findings was the increased frequency of HAE attacks among participants with HAE-nl-C1INH. Participants with HAE-nl-C1INH were significantly more likely to report experiencing multiple attacks per week; 47% (21 of 45) reported ≥ 52 to < 365 attacks per year. In contrast, participants with HAE Type 1/2 reported less frequent episodes; 39% (17 of 44) reported ≥ 12 to < 52 attacks, which aligns with a previous report of an average of 26.9 attacks per year in patients with HAE [5]. We recognized that both groups may have included participants who were newly diagnosed and not receiving treatment at the time of their participation in this survey, which may have affected their reported frequency of attacks. Moreover, participants with HAE-nl-C1INH were more likely to experience orofacial-laryngeal swelling. This is particularly concerning given the challenges of obtaining an accurate and timely diagnosis for participants with HAE-nl-C1INH [1, 3]. Participants with HAE-nl-C1INH more frequently reported receiving a diagnosis at ≥ 18 years of age, whereas participants with HAE Type 1/2 had greater odds of being diagnosed at < 18 years of age. These results align with those from an international consensus of 31 HAE-nl-C1INH global experts that suggested that patients with HAE-nl-C1INH experience onset of swelling at a later age and a greater prevalence of orofacial-laryngeal swelling than abdominal attacks compared with patients with HAE-C1-INH [10]. The varied diagnostic patterns in these patient-reported outcomes align with results from a prior survey study in which clinicians who treat patients with HAE reported diagnostic delays among patients with HAE-nl-C1INH [9]. These delays may be attributable to the lack of clear biomarkers and diagnostic process as well as the heterogeneity of clinical presentation [1, 9].

Another key finding was the higher frequency of medical visits among participants with HAE-nl-C1INH. Notably, these individuals were more likely to receive care from immunologists, whereas participants with HAE Type 1/2 more frequently received care from allergists. This may reflect differing diagnostic, referral, and management approaches for HAE subtypes based on provider specialty in this underrecognized patient group. In addition to differences in disease severity, the greater frequency of specialist visits in patients with HAE-nl-C1INH may reflect the diagnostic difficulties associated with HAE without C1INH deficiency. Given the higher frequency of attacks and increased medical visits reported by these participants, there is a clear need for improved management strategies, including tailored prophylactic and on-demand therapies.

The impact of HAE on socioeconomic outcomes was also evident in this study. Participants with HAE-nl-C1INH were less likely to work full-time and reported lower annual incomes compared with those with HAE Type 1/2. Furthermore, overall HRQoL, along with the domains of feeling and concerns, was significantly worse in participants with HAE-nl-C1INH than in those with HAE Type 1/2. These results align with reports from a prior survey study in which patient-reported HRQoL was lower in patients with HAE-nl-C1INH than in those with HAE Type 1/2 [13]. It is possible that patients with HAE-nl-C1INH may experience worse HRQoL in part due to the pathophysiological ambiguity of a rare disease, particularly when patients with different subtypes of HAE-nl-C1INH may experience different treatment responses to various therapies [10, 14]. The unpredictable and severe nature of their symptoms likely contributes to work-related disruptions and financial instability, potentially leading to low socioeconomic status [1]. Importantly, socioeconomic status can influence stress levels, comorbidity burden, and access to specialty care [15–17]. Therefore, an alternative explanation is that those in lower income groups may have reduced access to early diagnosis and prophylactic therapy, thereby increasing disease burden. These findings highlight the need for comprehensive support strategies that extend beyond clinical management to include workplace accommodations and mental health support.

The increased burden of concomitant conditions among participants with HAE-nl-C1INH also warrants attention. Autoimmune disorders, asthma, anxiety, and depression were all reported at higher numerical rates in this group than in those with HAE Type 1/2. Although the mechanisms behind these associations remain unclear, some data suggest that subtle inflammatory modulation—through mast cells and interleukin-driven cytokine networks influencing vascular permeability and nociceptive signaling—may shape symptom perception [18–20]. It is also possible that patients with HAE-nl-C1INH may have greater diagnostic uncertainty and/or medical surveillance, leading to more frequent comorbidity labeling by physicians. Regardless of the etiology, the presence of multiple comorbidities may further compound the challenges faced by these patients, contributing to increased health care needs and diminished quality of life.

Several limitations should be considered when interpreting these findings. The unadjusted comparisons presented here are limited. Ideally, with sufficient sample size and data from future epidemiologic studies to identify confounders, these factors could be incorporated as interaction variables in the analysis, enabling a more robust comparison between populations. As a survey-based study, recall and response bias may have influenced the results, potentially leading to overreporting or underreporting of symptoms and health care utilization. Given the unpredictable and episodic nature of HAE, the 3-month recall period may have captured periods of unusual calm or flare, which could have potentially contributed to observed differences between groups. Future research could include longitudinal follow-up to help distinguish sustained attack burden from transient fluctuations. Furthermore, self-reported data were not confirmed by medical records, which may affect the accuracy of reported diagnoses, comorbidities, and treatment patterns. Participants self-reported their type of HAE without laboratory confirmation or direct diagnostic validation, thereby providing a risk of categorial misclassification that may have influenced the measured differences between the HAE Type 1/2 and HAE-nl-C1INH groups. Future studies should verify HAE type using medical records. Additionally, rather than expanding recruitment efforts elsewhere, participants were invited from a patient support program associated with their prescription HAE treatment, as this approach was the most feasible to achieve the sample size required for this rare disease. Individuals enrolled in specialty pharmacy support programs may be more engaged with health care services and may be more likely to have access to specialty care [21]. Because the impact of this sampling was the same for participants with HAE-nl-C1INH and those with HAE Type 1/2, this influence would have been distributed evenly across groups and is unlikely to have influenced the comparative results between groups. Future research should aim to improve inclusive sampling, such as partnering with patient advocacy networks or applying probabilistic sampling approaches to outpatient claims databases. Finally, this study was conducted during the COVID-19 pandemic, which likely influenced health care access, physician availability, and patient behavior. Nonetheless, given the rarity of HAE and the paucity of patient-reported data, this study provides valuable insights into the lived experiences of patients with different HAE subtypes [3].

## Conclusions

In conclusion, this study highlights the substantial burden experienced by patients with HAE-nl-C1INH compared with those with HAE Type 1/2, reflecting a more intense disease phenotype. The increased frequency of attacks, including orofacial-laryngeal swelling, greater health care utilization, higher prevalence of concomitant conditions, worse quality of life, and heightened socioeconomic challenges among patients with HAE-nl-C1INH highlight a substantial unmet need in this population. These findings underscore the need for improved diagnostic and therapeutic strategies tailored to the severity and intensity of disease. They also highlight the importance of an improved understanding of the prevalence, etiology, and pathophysiology of HAE-nl-C1INH. Future research should focus on optimizing treatment approaches and addressing the broader health disparities faced by this patient population.

## Data Availability

The datasets generated for this study are not publicly available, as it may be possible to link anonymized patient data back to individual patients due to the rarity of this disease. Reasonable requests for specific data fields or variables can be sent to the corresponding author for consideration.
